# A systematic review and meta-analysis of acupuncture in aspiration caused by post-stroke dysphagia

**DOI:** 10.3389/fneur.2024.1305056

**Published:** 2024-06-10

**Authors:** Hongwei Li, Jie Li, Xu Wang, Zhilong Zhang

**Affiliations:** ^1^Tianjin University of Traditional Chinese Medicine, Tianjin, China; ^2^Tianjin Academy of Traditional Chinese Medicine Affiliated Hospital, Tianjin, China

**Keywords:** acupuncture, stroke, dysphagia, aspiration, systematic review, meta-analysis

## Abstract

**Objective:**

This systematic review and meta-analysis aims to systematically evaluate the effectiveness and safety of acupuncture in the treatment of aspiration caused by post-stroke dysphagia.

**Methods:**

A computer search was conducted in nine databases, including the China National Knowledge Infrastructure (CNKI), China Science and Technology Journal (VIP), Wan-fang Database, China Biomedical Literature Database (CBM), PubMed, Web of Science, Cochrane Library, Embase, and Chinese Clinical Trial Registry (ChiCTR), from their inception until April 2024. Clinical randomized controlled trials comparing acupuncture combined therapy or single therapy with control interventions for the treatment of aspiration caused by post-stroke dysphagia were included. The primary outcome measure was the Penetration Aspiration Scale (PAS), and secondary outcome measures included the overall effective rate, video fluoroscopic swallowing study (VFSS), and hyoid bone displacement. The statistical analysis was performed using RevMan 5.3 and Stata 16.0.

**Results:**

A total of 16 articles involving 1,284 patients were included. The meta-analysis results showed that acupuncture combined therapy or single therapy was more effective in improving PAS scores compared to conventional rehabilitation therapy or balloon dilation of the catheter [WMD = −1.05, 95% CI (−1.30, −0.80), *Z* = 0.82, *p* = 0.00 < 0.05]. It was also more effective in improving VFSS scores [WMD = 1.32, 95% CI (0.08, 2.55), *Z* = 2.09, *p* = 0.04 < 0.05] and hyoid bone displacement [WMD = 2.02, 95% CI (0.86, 3.18), *Z* = 3.41, *p* = 0.00 < 0.05]. Additionally, acupuncture had a higher overall effective rate [WMD = 1.21, 95% CI (1.14, 1.29), *Z* = 5.76, *p* = 0.00 < 0.05] and a lower incidence of adverse events. Sensitivity analysis indicated that the literature had minimal impact on the results, and bias tests showed no publication bias.

**Conclusion:**

Acupuncture combined therapy and acupuncture single therapy can effectively improve aspiration caused by post-stroke dysphagia with a low incidence of adverse events. However, due to the low quality of the included literature, more high-quality randomized controlled trials are still needed to confirm the effectiveness and safety of acupuncture in the treatment of aspiration caused by post-stroke dysphagia.

**Systematic review registration:**

https://www.crd.york.ac.uk/prospero/display_record.php?ID=CRD42023462707, identifier CRD42023462707

## 1 Introduction

Aspiration refers to the phenomenon of the entry of oral or gastric contents below the glottis into the respiratory tract. It is the most severe and immediate consequence of post-stroke dysphagia (PSD) (1) and one of the leading factors causing pneumonia (2). Following aspiration, patients immediately experience irritative coughing, dyspnea, and even asthma, known as dominant aspiration (DA). Silent aspiration (SA) refers to the absence of external signs such as coughing within 1 min after aspiration, without symptoms of irritative coughing or dyspnea. The incidence of aspiration in PSD ranges from 15 to 54% (2, 3), with approximately 68% being SA, which is difficult to detect through non-imaging examinations (3, 4), leading to potential underdiagnosis. Hence, the actual incidence of SA may be higher (5). Aspiration can result in malnutrition, pneumonia, and even suffocation (6). Studies have shown that the occurrence rate of pneumonia in patients with aspiration is 11 times higher than in other patients (7), and the overall 30 day mortality rate of aspiration pneumonia is 21–30% (8), significantly affecting patient prognosis, prolonging hospital stays, and increasing treatment costs (9).

Clinical guidelines in the United States (10), the United Kingdom (11), Germany (12), and other countries recommend that acute stroke patients should undergo swallowing screening by trained healthcare professionals within 4 h of hospital admission or before taking any solid, liquid medications, or food. Many screening criteria include the presence of coughing symptoms (5). However, SA is more covert clinically, as patients may not exhibit symptoms such as coughing. Therefore, SA cannot be easily determined through clinical swallowing screening and requires imaging examinations. Currently, video fluoroscopic swallowing study (VFSS) is considered the gold standard for diagnosing aspiration both domestically and internationally (13, 14). It utilizes natural eating methods to create visual images of tongue movement, food transmission in the pharynx, elevation of the hyoid bone and larynx, and movement of the soft palate and epiglottis. By observing the flow of contrast agent from the oral cavity to the cervical esophagus and determining the depth of contrast agent entry into the airway, penetration or aspiration can be accurately assessed, reducing missed diagnoses (15). The Penetration Aspiration Scale (PAS) is an assessment scale derived from VFSS, which allows clinicians to visually understand the presence of aspiration in stroke patients through numerical scores. It provides a reliable quantitative indicator for evaluating the severity of aspiration and serves as a reliable tool for swallowing function assessment (16).

The mechanism of aspiration caused by PSD remains unclear and may be related to weakened pharyngeal reflexes, weakened pharyngeal muscle strength, and decreased coordination due to nerve damage. It could also be associated with reduced pharyngeal sensation, impaired reflex coughing ability, or low levels of P substance and dopamine (3). Modern medical treatments for post-stroke dysphagia and aspiration include swallowing training, local muscle stimulation, dietary improvements, and changes in eating positions. Although these methods have proven effectiveness, they require evaluation by specialized rehabilitation therapists, and their efficacy may vary among different populations (1). Therefore, the discovery of safe and effective treatment methods holds immense clinical significance.

Acupuncture, as a simple, environmentally friendly, and cost-effective treatment method, has been supported by a large number of clinical studies and evidence-based medicine to effectively treat post-stroke dysphagia (17, 18). The Chinese Expert Consensus on Swallowing Disorders and Nutritional Management in Stroke Patients also recommends acupuncture as a Class A treatment method (19). However, some studies on aspiration caused by PSD have used non-imaging examinations as outcome measures (20), or the outcome measures lacked a quantitative scale for analyzing the severity of aspiration (21), which cannot demonstrate the efficacy of acupuncture for SA. Therefore, this study conducted a meta-analysis to evaluate the efficacy and safety of acupuncture in the treatment of aspiration caused by post-stroke dysphagia, aiming to provide evidence-based medicine support for its effectiveness.

## 2 Methods

This study has been registered in PROSPERO (registration number: CRD42023462707), and the registration details can be obtained from https://www.crd.york.ac.uk/prospero/. The reporting of study results follows the Preferred Reporting Items for Systematic Reviews and Meta-Analyses (PRISMA) 2020 checklist (see Appendix 1).

### 2.1 Data sources and search strategy

A computer search was conducted in nine databases, including CNKI, VIP, WF, SinoMed, PubMed, Web of Science, Cochrane Library, EMbase, and ChiCTR, from their inception until April 2024. Chinese subject terms and free terms were determined using the subject term search function in SinoMed, while English subject terms and free terms were determined using the MeSH Database in PubMed. The Chinese subject terms included “卒中” (stroke), “吞咽障碍” (deglutition disorders), “针刺” (acupuncture), and “随机对照试验” (randomized controlled trial). The English subject terms included “stroke,” “Deglutition Disorders,” “acupuncture,” and “randomized controlled trial.” There were no language restrictions on the literature search. The specific search terms and strategies are detailed in Appendix 2.

### 2.2 Inclusion criteria

1. Study type: randomized controlled trials.

2. Study population: patients diagnosed with stroke according to the guidelines for the prevention of stroke issued by the American Heart Association and the American Stroke Association (22) or the diagnostic criteria for stroke in the Chinese Guidelines for the Prevention and Treatment of Stroke (2021 edition) (23). The patients should also meet the diagnostic criteria for swallowing disorders established by the European Stroke Organization and the European Society for Swallowing Disorders (24).

3. Intervention: the experimental group received acupuncture therapy alone or in combination with other therapies compared to the control group, which received any therapy other than acupuncture.

4. Primary outcome measure: Rosenbek Penetration Aspiration Scale (PAS). Secondary outcome measures included overall effective rate, video fluoroscopic swallowing study (VFSS), and hyoid bone displacement. The studies had to include the primary outcome measure and at least one of the secondary outcome measures.

### 2.3 Exclusion criteria

1. Subjects with other conditions affecting swallowing function, such as tumors, myasthenia gravis, Parkinson’s syndrome, Guillain-Barré syndrome, etc.

2. Subjects with other life-threatening diseases, such as severe coronary heart disease, heart failure, severe pneumonia, etc.

3. Duplicate publications.

4. Literature with incomplete trial data.

5. Literature for which the full text could not be obtained.

### 2.4 Literature screening and data extraction

**Figure 1:**
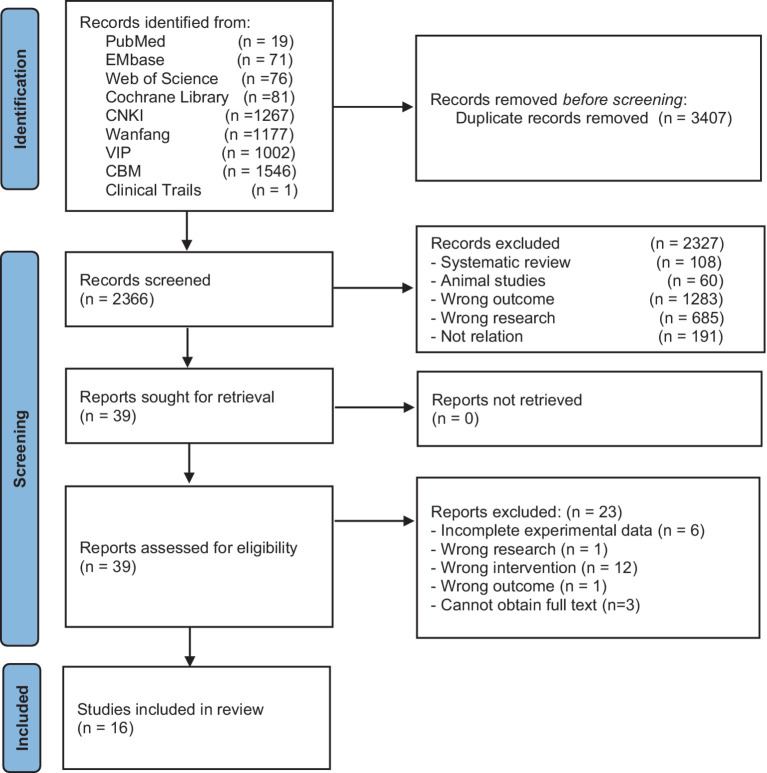
Flowchart of the literature search process for acupuncture treatment of aspiration caused by post-stroke dysphagia.

Following the methods for including studies described in the Cochrane Handbook for Systematic Reviews of Interventions (25), the search results from each database were imported into the reference management software NoteExpress. The screening process was conducted based on the inclusion and exclusion criteria, as shown in Figure 1. The eligible literature was then organized and recorded in Excel, including information such as study authors, publication dates, sample sizes, gender distribution, average age, intervention measures, treatment frequency and duration, outcome measures, and adverse events. Two independent reviewers conducted the literature screening and data extraction, with cross-checking of the results. If any discrepancies arose during the study selection or data extraction process, a third reviewer intervened to make a judgment.

When outcome measures were presented as medians (*m*), first quartile (*q*1), third quartile (*q*3), and/or minimum (*a*) and maximum (*b*) values, they were transformed into mean ± standard deviation using statistical conversion formulas (26, 27).


$$S\approx \frac{b-a}{2\Phi^{-1}\left(\frac{n-0.375}{n+0.25}\right)}\;S\approx \frac{q_{3}-q_{1}}{2\Phi^{-1}\left(\frac{0.75n-0.125}{n+0.25}\right)}$$

$$\begin{array}{l} \bar{X}\left(w\right)\approx \left(\frac{4}{4+n^{0.75}}\right)\frac{a+b}{2}+\left(\frac{n^{0.75}}{4+n^{0.75}}\right)m\;\\ \bar{X}\left(w\right)\approx \left(0.7+\frac{0.39}{n}\right)\frac{q_{1}+q_{3}}{2}+\left(0.3-\frac{0.39}{n}\right)m \end{array}$$

When an outcome measure is presented in the form of multiple subgroups, the data from the subgroups are combined using the formula provided in the Cochrane Handbook for Systematic Reviews of Interventions (28).


$$\mathrm{Sample}\;\mathrm{size}\;\left(\mathrm{Combined}\;\mathrm{groups}\right)=N1+N2.$$

$$\mathrm{Mean}\;\left(\mathrm{Combined}\;\mathrm{groups}\right)=\frac{N_{1}M_{1}+N_{2}M_{2}}{N_{1}+N_{2}}$$

$$\begin{array}{l} SD\;\left(\mathrm{Combined}\;\mathrm{groups}\right)=\\ \;\sqrt{\frac{\left(N_{1}-1\right){SD}_{1}^{2}+\left(N_{2}-1\right){SD}_{2}^{2}+\frac{N_{1}N_{2}}{N_{1}+N_{2}}\left(M_{1}^{2}+M_{2}^{2}-2M_{1}M_{2}\right)}{N_{1}+N_{2}-1}} \end{array}$$

### 2.5 Quality assessment

All studies included in this research were randomized controlled trials. Therefore, the Risk of Bias 2 (RoB 2) tool provided by the Cochrane Collaboration, as recommended by the GRADE evidence grading system, was utilized to assess the risk of bias in the included studies (29). The assessment criteria included random sequence generation, allocation concealment, blinding, completeness of outcome data, selective reporting, and other biases. The included literature was categorized as having low, unclear, or high risk of bias based on the assessment criteria. Two trained reviewers (JL and XW) independently assessed the included studies using RoB 2 and calculated the intra-class correlation coefficient (ICC) to conduct a consistency test. If the consistency reached at least 80%, a formal assessment was conducted. Any discrepancies were resolved through discussion with a third reviewer (ZZ).

### 2.6 Statistical analysis

Statistical analysis was conducted using RevMan 5.3 and Stata 16.0. The analysis included tests for baseline consistency, heterogeneity, sensitivity analysis, and publication bias assessment using funnel plots when the number of included studies exceeded 5. For continuous variables, mean differences (MD) were used as the effect size, and for binary variables, risk ratios (RR) were used as the effect size. The 95% confidence intervals (CI) were calculated, and a *p*-value <0.05 was considered statistically significant. The criteria for heterogeneity testing were based on the Cochrane Handbook for Systematic Reviews of Interventions 5.0.2 (30). Heterogeneity was assessed by calculating the *I*^2^ statistic and the *p*-value from the *Q* test. *I*^2^ values between 0 and 40% indicated low heterogeneity, 30 to 60% indicated moderate heterogeneity, 50 to 90% indicated substantial heterogeneity, and *I*^2^ > 75% indicated high heterogeneity. Additionally, a *p*-value <0.1 from the *Q* test indicated significant heterogeneity. In this study, a fixed-effects model was used for analysis when *I*^2^ ≤ 50% and *p* ≥ 0.1, and a random-effects model was used for analysis when *I*^2^ > 50% and *p* < 0.1. Meta-regression was conducted to identify the sources of heterogeneity. Sensitivity analysis was performed using the one-by-one exclusion method, and if the pooled effect size fell outside the 95% confidence interval, it was considered to have a strong impact on the study results. Bias assessment was conducted by plotting a funnel plot and calculating the bias *p*-value, where a *p*-value >0.05 indicated no publication bias.

## 3 Results

### 3.1 Literature selection

A total of 5,773 articles were identified through the database search in nine databases (CNKI = 1,267, VIP = 1,002, WF = 1,711, CBM = 1,546, PubMed = 19, Web of Science = 76, Cochrane Library = 81, Embase = 71, Clinical Trials = 1). After removing duplicates (*n* = 3,407), 2,327 articles were excluded based on the title and abstract. Full-text screening resulted in the exclusion of 23 articles, and finally, 16 articles were included (31–46). The flowchart of the literature selection process is shown in Figure 1.

### 3.2 Characteristics of included studies

The 16 included studies were all single-center randomized controlled trials conducted in China, involving a total of 1,284 cases, with 642 cases in the experimental group and 642 cases in the control group. Three studies were master’s theses (40, 42, 45), and the remaining 13 were journal articles. The average age of the patients ranged from 56 to 71 years. The average duration of stroke was less than 1 day in one study (36), more than 3 months in one study (44), and unreported in one study (41), while the average duration in the remaining studies ranged from 15 days to 75 days. The proportion of stroke types was not reported in two studies (39, 41), one study included only ischemic stroke patients (37), and the remaining studies included more ischemic stroke patients than hemorrhagic stroke patients. Three studies used acupuncture combined therapy (41, 43, 46), while the remaining studies used acupuncture monotherapy. One study used electroacupuncture (35), one study used auricular acupressure (33), and the remaining studies used conventional acupuncture. All studies compared the PAS scores, nine studies compared the overall effective rate (31–33, 36, 39, 40, 42, 44, 46), four studies compared VFSS scores (36, 41, 42, 44), and three studies compared hyoid bone displacement (38, 40, 43). The basic characteristics of the included studies are presented in Table 1.

**Table 1:** Basic characteristics of included studies on acupuncture treatment for aspiration caused by post-stroke dysphagia.

Author and year of publication	Course	Frequency of treatment	Adverse events		Type of stroke (Cases)		Interventions	Course (Days)	Age (Years)	Finale index
					Haemorrhage	Ischemia				
Cao 2023 (31)	4 weeks	6 times a week	Not mentioned	T	13	17	Acupuncture	34.0 ± 22.1	61 ± 8	PAS, Overall effective rate
				C	12	18	NMES	36.8 ± 47.4	59 ± 7	
Chen 2018 (32)	4 weeks	5 times a week	Not mentioned	T	9	21	Acupuncture	39.20 ± 12.61	62.90 ± 10.04	PAS, Overall effective rate
				C	6	24	Swallowing training	36.83 ± 14.6	63.00 ± 9.83	
Chen 2022 (33)	2 weeks	7 times a week	Not mentioned	T	11	29	Auriculopathic compression	62.05 ± 7.08	62.05 ± 7.08	PAS, Overall effective rate
				C	18	22	Swallowing training	59.70 ± 5.80	59.70 ± 5.80	
Huang 2021 (34)	3 weeks	5 times a week	Not mentioned	T	8	13	Acupuncture	64.68 ± 38.71	70.90 ± 7.09	PAS
				C	7	14	NMES	64.68 ± 44.38	71.33 ± 6.39	
Kang 2023 (35)	3 weeks	5 times a week	Not mentioned	T	3	12	Electroacupuncture	26.87 ± 29.57	61.80 ± 12.45	PAS
				C	6	24	NMES+Swallowing training	41.20 ± 33.95	63.44 ± 10.83	
Li 2017 (36)	8 weeks	1 time per day	Not mentioned	T	17	23	Acupuncture	0.35 ± 0.72	63.6 ± 11.34	PAS, FESS, overall effective rate
				C	33	37	Swallowing training	0.37 ± 0.42	63.57 ± 11.08	
Li 2019 (37)	4 weeks	6 times a week	Not mentioned	T	0	40	Acupuncture	16.9 ± 7.1	61.9 ± 7.9	PAS
				C	0	40	Swallowing training	18.5 ± 8.1	63.6 ± 6.9	
Lin 2021 (38)	4 weeks	5 times a week	Not mentioned	T	17	28	Acupuncture	47.50 ± 9.90	60.40 ± 9.00	PAS, Hyoid bone displacement
				C	14	31	Rtms	46.80 ± 9.70	59.70 ± 9.20	
Lin 2022 (39)	4 weeks	5 times a week	None	T	Not mentioned	Not mentioned	Acupuncture	50.1 ± 20.9	61.00 ± 14.00	PAS, Overall effective rate
				C	Not mentioned	Not mentioned	Swallowing training	48.4 ± 24.9	60 ± 13	
Mo 2021 (40)	4 weeks	6 times a week	None	T	16	24	Acupuncture	73.65 ± 6.41	68.28 ± 0.67	PAS, Hyoid bone displacement, Overall effective rate
				C	18	22	BDC	74.73 ± 6.80	66.73 ± 0.58	
Pan 2023 (41)	3 weeks	5 times a week	Not mentioned	T	Not mentioned	Not mentioned	Acupuncture + Mirror therapy	Not mentioned	62.87 ± 5.43	PAS, FESS
				C	Not mentioned	Not mentioned	Mirror therapy	Not mentioned	65.87 ± 6.35	
Wang 2021 (42)	4 weeks	6 times a week	Not mentioned	T	9	22	Acupuncture	41.32 ± 37.01	63.58 ± 10.29	PAS, FESS, Overall effective rate
				C	11	20	Swallowing training	36.06 ± 37.73	63.90 ± 10.19	
Wu 2022 (43)	4 weeks	5 times a week	Not mentioned	T	35	36	Acupuncture + NMES + BDC	38.01 ± 7.84	57.65 ± 5.73	PAS, Hyoid bone displacement
				C	37	34	NMES+BDC	32.27 ± 7.49	56.82 ± 6.33	
Xia 2020 (44)	3 weeks	5 times a week	Not mentioned	T	12	25	Acupuncture	90 ± 48	60.2 ± 12.3	PAS, FESS, Overall effective rate
				C	13	24	Swallowing training	93 ± 48	66.1 ± 10.5	
Yang 2022 (45)	4 weeks	6 times a week	None	T	13	21	Acupuncture	52.4 ± 17.2	65.5 ± 3.60	PAS
				C	15	19	NMES	51.20 ± 16.70	66.60 ± 3.20	
Zhou 2018 (46)	4 weeks	5 times a week	Not mentioned	T	8	20	Acupuncture + DPNS	15 ± 8	66.7 ± 13.6	PAS, Overall effective rate
				C	9	19	DPNS	17 ± 7	67.0 ± 12.9	

T, intervention group; C, control group; BDC, balloon dilatation Catheter; DPNS, deep pharyngeal neuromuscular stimulation; rTMS, high-frequency repetitive transcranial magnetic stimulation; NMES, neuromuscular electrical stimulation.

### 3.3 Risk of bias in the included studies

The ICC value for the RoB 2 assessment by the two reviewers was 0.848, indicating excellent consistency.

**Figure 2:**
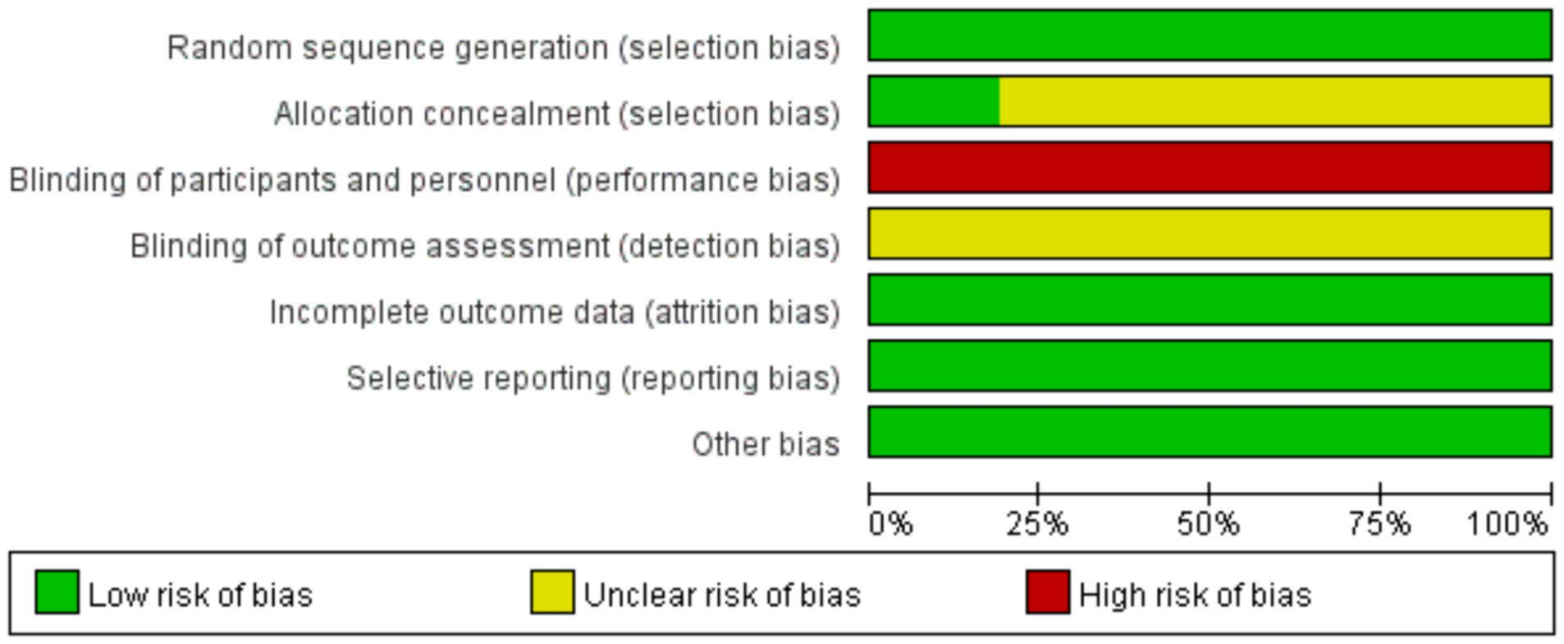
Risk of bias analysis.

**Figure 3:**
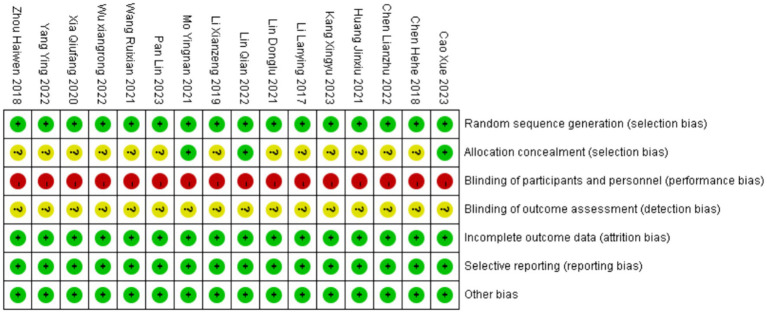
Summary of risk of bias.

All 16 studies included in the analysis were randomized controlled trials. Among them, 10 studies (32–35, 40, 42–46) utilized random number tables for grouping, one study (36) did not specify the method of randomization, and three studies (31, 39, 44) achieved allocation concealment through opaque envelopes. Two studies (40, 45) experienced participant dropout, with explanations provided for the reasons, and the dropout rates were <10%. The baseline data between the two groups remained comparable, thus not affecting the integrity of the study results. Since all studies obtained informed consent from the participants, blinding was not implemented in any of the studies. Overall, 13 studies (32–38, 42–46) were categorized as having a high risk of bias, while 3 studies (31, 39, 40) were categorized as having an unclear risk of bias. The quality assessment results of the literature are summarized in Figures 2, 3.

### 3.4 Meta-analysis results

#### 3.4.1 Penetration aspiration scale

A total of 16 studies (31–46) involving the PAS scale were included, with a total of 1,284 cases. The baseline consistency analysis showed low heterogeneity in the baseline effects of the PAS scale between the experimental and control groups (*I*^2^ =0%<50%, and *p* = 0.82>0.1 from the Q test). Therefore, a fixed-effects model was selected to combine the effect sizes. The combined effect size for the baseline effects was 0.08 (*z* = 1.73, *p* = 0.08>0.05), indicating no statistically significant difference in the baseline period. Subsequent meta-analysis could be performed (see Supplementary Figure S1).

**Figure 4:**
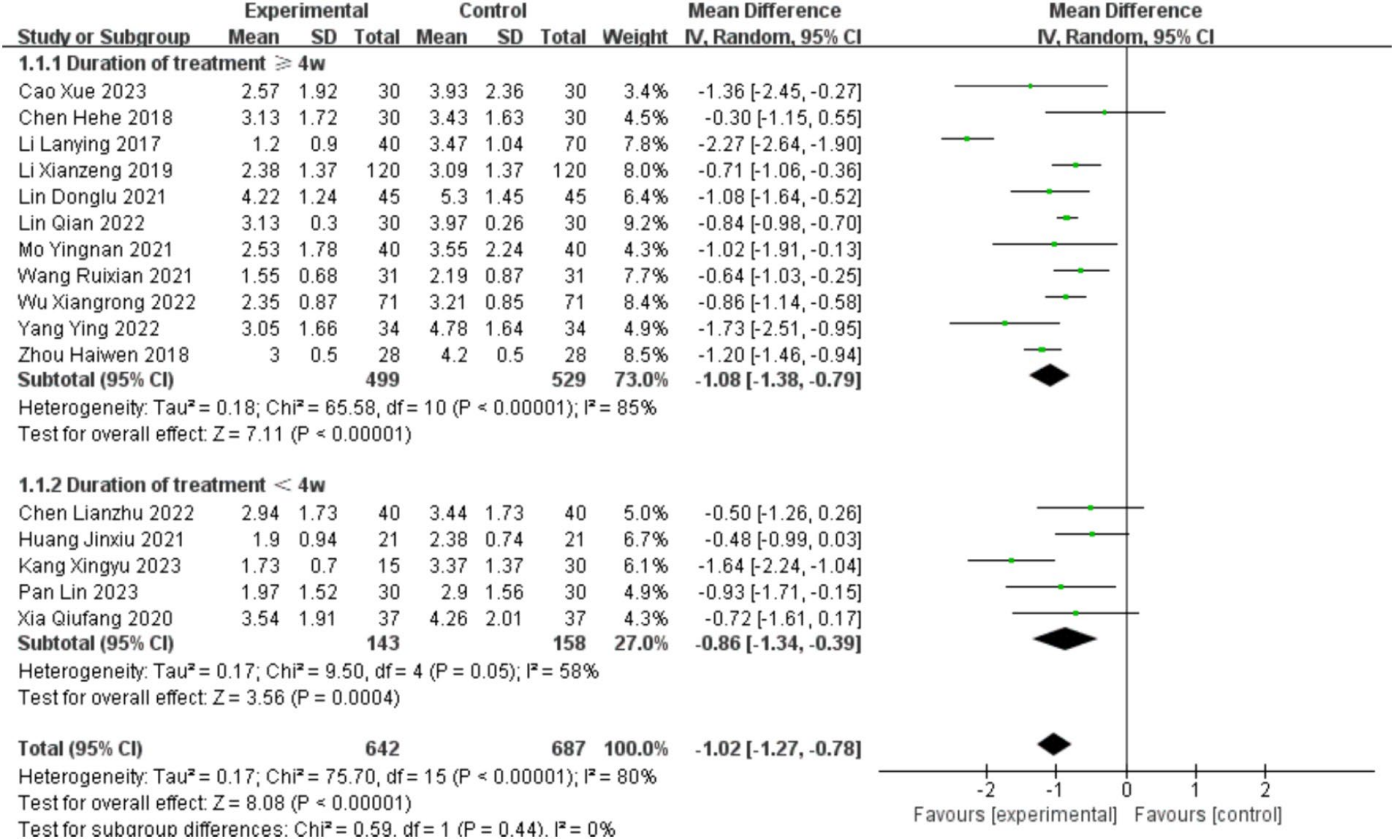
Forest plot of PAS scores.

The analysis was stratified into two subgroups based on treatment duration: ≥4 weeks and <4 weeks. There was no statistically significant difference between the subgroups (*p* = 0.44, >0.05, *I*^2^ = 0%). Furthermore, even after subgroup analysis, there was still significant heterogeneity in the PAS scale scores (*I*^2^ = 80 > 50%, and the *p* value of the *Q* test was 0.000 < 0.05). Therefore, a random-effects model was chosen for the meta-analysis (see Figure 4).

Due to the high heterogeneity in the combined effect size, meta-regression was conducted to analyze the sources of heterogeneity based on factors such as publication year, intervention measures (acupuncture monotherapy/acupuncture combined therapy), average duration of stroke (0–1 month/1–2 months/2–3 months), and treatment duration (less than 4 weeks/4 weeks or more). The results showed that none of these factors significantly contributed to the heterogeneity (*p* > 0.05) (see Table 2).

**Table 2:** Meta-regression of PAS scores.

_it	Coef.	Std. Err.	*t*	*P* > |*t*|	[95% Conf.Interval]	
Year	−0.02647	0.091943	−0.29	0.779	−0.22883	0.1758981
Interventions	−0.12439	0.356066	−0.35	0.733	−0.90808	0.6593082
Course	0.099043	0.373485	0.27	0.796	−0.72299	0.9210771
Average course of illness	0.286838	0.201132	1.43	0.182	−0.15585	0.7295267
_cons	51.80965	185.7511	0.28	0.785	−357.026	460.6451

**Figure 5:**
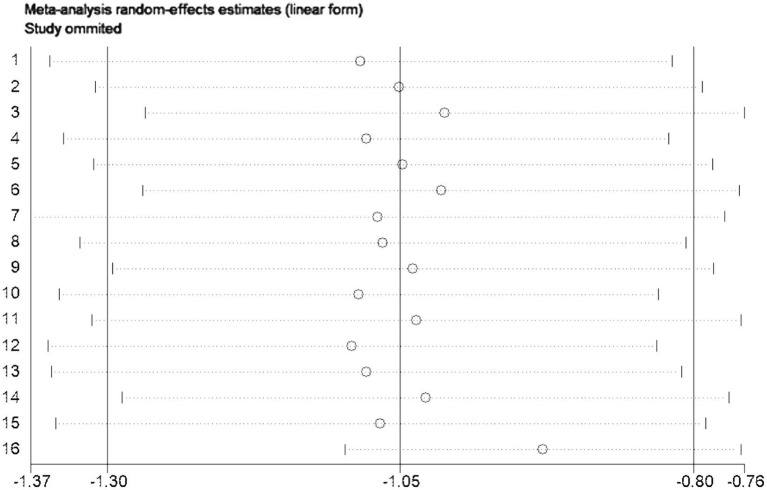
Sensitivity analysis of PAS scores.

Sensitivity analysis using the one-by-one exclusion method was performed for the studies involving the PAS scale, and no study was found to have a strong impact on the results (see Figure 5).

**Figure 6:**
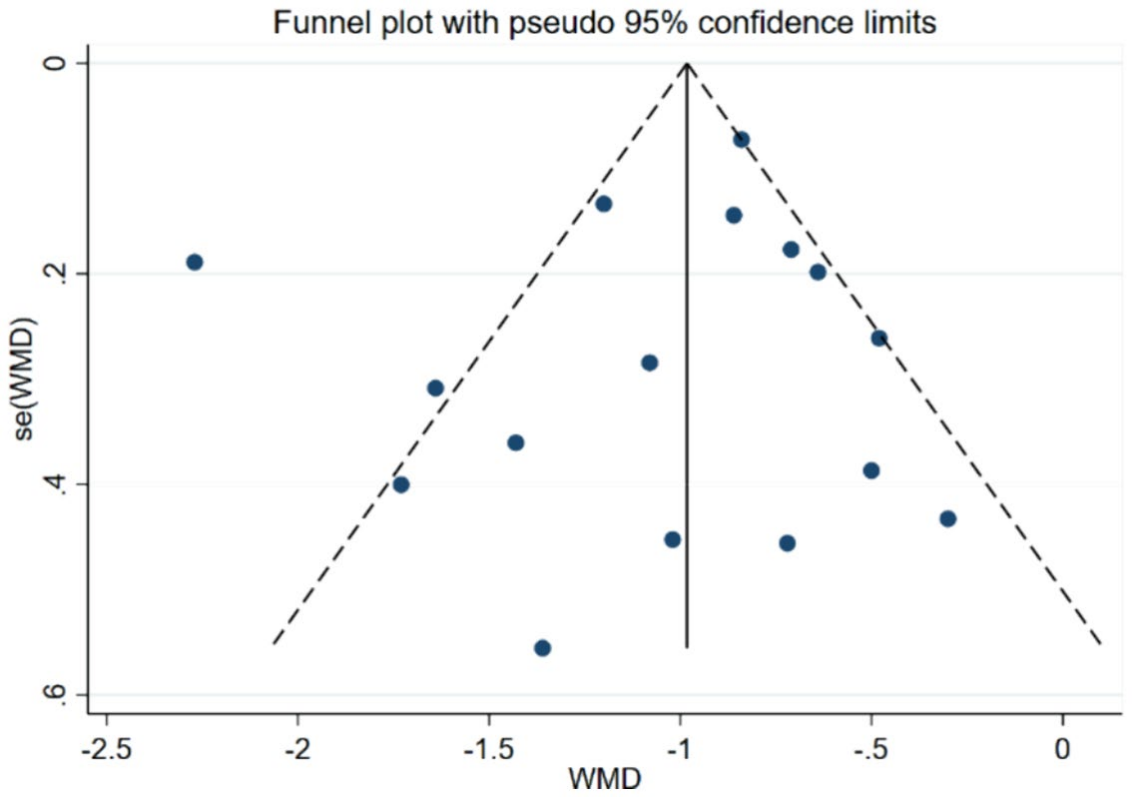
Funnel plot of PAS scores.

Bias assessment was conducted for the studies involving the PAS scale, and the results showed a *p*-value of 0.519 > 0.05, indicating no publication bias in this study (see Figure 6).

#### 3.4.2 Overall effective rate

Nine studies (31–33, 36, 39, 40, 42, 44, 46) involved statistical analysis of total effective rate, totaling 612 cases included. Based on treatment duration, the analysis was divided into two subgroups: ≥4 weeks and <4 weeks. There was no statistically significant difference between the subgroups (*p* = 0.25, >0.05, *I*^2^ = 25.6%). The heterogeneity of the total effective rate was low (*I*^2^ = 0 < 50%, and the *p* value of the *Q* test was 0.92 > 0.1), so a fixed-effects model was chosen for the meta-analysis.

**Figure 7:**
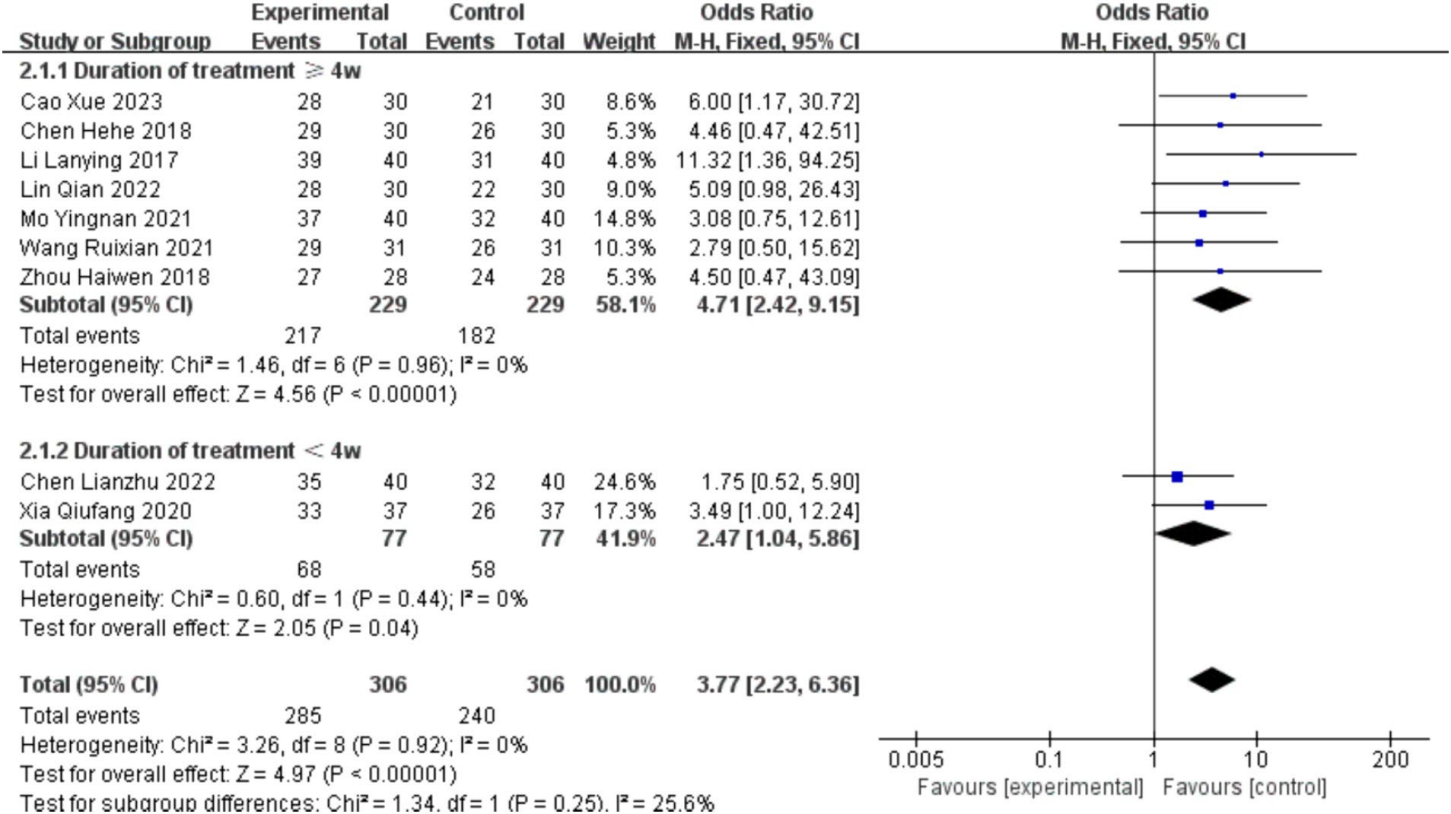
Forest plot of overall effective rate.

For studies with treatment duration ≥4 weeks, the combined effect size was 4.71 (2.42, 9.15), and the effect size was significant (*Z* = 4.56, *p* = 0.00 < 0.05). For studies with treatment duration <4 weeks, the combined effect size was 2.47 (1.04, 5.86), and the effect size was significant (*Z* = 2.05, *p* = 0.04 < 0.05). The overall combined effect size was 3.77 (2.23, 6.36), and the effect size was significant (*Z* = 4.97, *p* = 0.00 < 0.05), indicating that the total effective rate in the intervention group was 3.77 times higher than that in the control group, demonstrating a significant intervention effect (see Figure 7).

**Figure 8:**
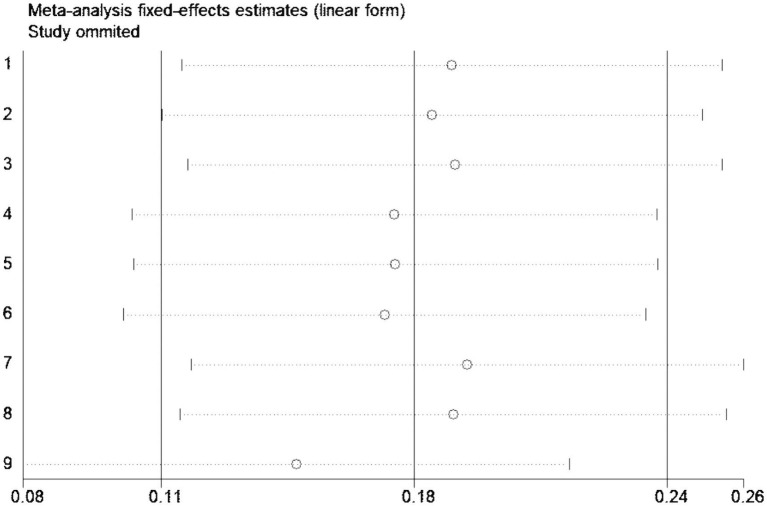
Sensitivity analysis of overall effective rate.

Sensitivity analysis using the one-by-one exclusion method was performed for the studies involving the overall effective rate, and no study was found to have a strong impact on the results (see Figure 8).

**Figure 9:**
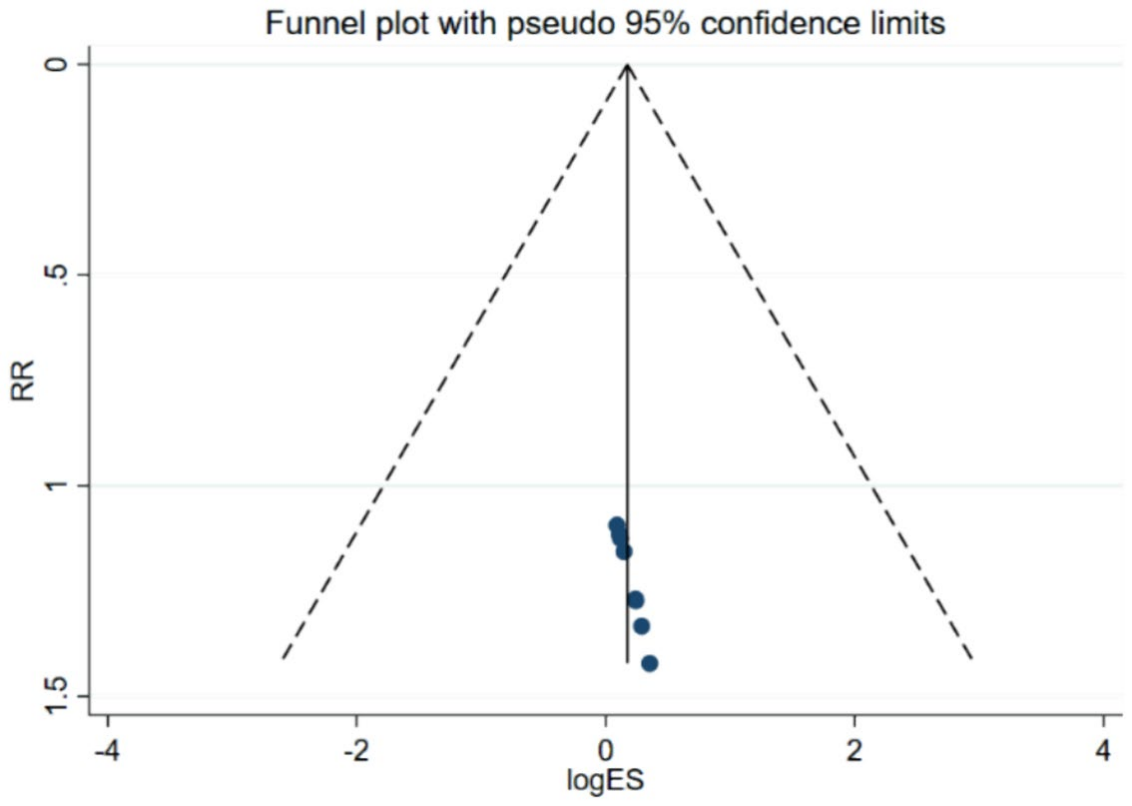
Funnel plot of overall effective rate.

Bias assessment was conducted for the studies involving the overall effective rate, and the results showed a *p*-value of 0.388 > 0.05, indicating no publication bias in this study (see Figure 9).

#### 3.4.3 Video fluoroscopic swallowing study

Four studies (36, 41, 42, 44) involved the statistical analysis of the VFSS scale, with a total of 306 cases included. The baseline consistency analysis showed low heterogeneity in the baseline effects of the VFSS scale between the experimental and control groups (*I*^2^ = 0% < 50%, and *p* = 0.92 > 0.1 from the *Q* test). Therefore, a fixed-effects model was used to combine the effect sizes. The combined effect size for the baseline effects was −0.08 (*Z* = 0.67, *p* = 0.50 > 0.05), indicating no statistically significant difference in the baseline period, allowing for subsequent meta-analysis (see Supplementary Figure S2).

**Figure 10:**
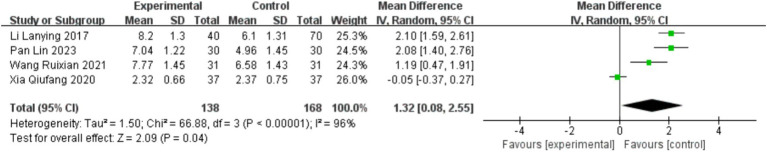
Forest plot of the VFSS scale.

According to the heterogeneity analysis, there was high heterogeneity in the effect sizes after treatment (*I*^2^ = 96% > 50%, and *p* = 0.000 < 0.05 from the *Q* test). Therefore, a random-effects model was used for the meta-analysis. The combined effect size was 1.32 (0.08, 2.55), and the effect size was significant (*Z* = 2.09, *p* = 0.04 < 0.05), indicating that the VFSS scale scores in the intervention group were significantly higher than those in the control group by 1.32. The intervention had a significant effect (see Figure 10).

Sensitivity analysis using the one-by-one exclusion method was performed for the studies involving the VFSS scale, and no study was found to have a strong impact on the results (see Supplementary Figure S3).

#### 3.4.4 Hyoid bone displacement

Three studies (38, 40, 43) involved the statistical analysis of hyoid bone displacement, with a total of 312 cases included. The studies were divided into two subgroups based on the distance of hyoid bone upward displacement and forward displacement. The baseline consistency analysis showed low heterogeneity in the baseline effects of hyoid bone displacement between the experimental and control groups (*I*^2^ = 0% < 50%, and *p* = 0.89 > 0.1 from the *Q* test). Therefore, a fixed-effects model was used to combine the effect sizes. The combined effect size for the baseline effects was 0.02 (*Z* = 0.12, *p* = 0.90 > 0.05), indicating no statistically significant difference in the baseline period, allowing for subsequent meta-analysis (see Supplementary Figure S4).

**Figure 11:**
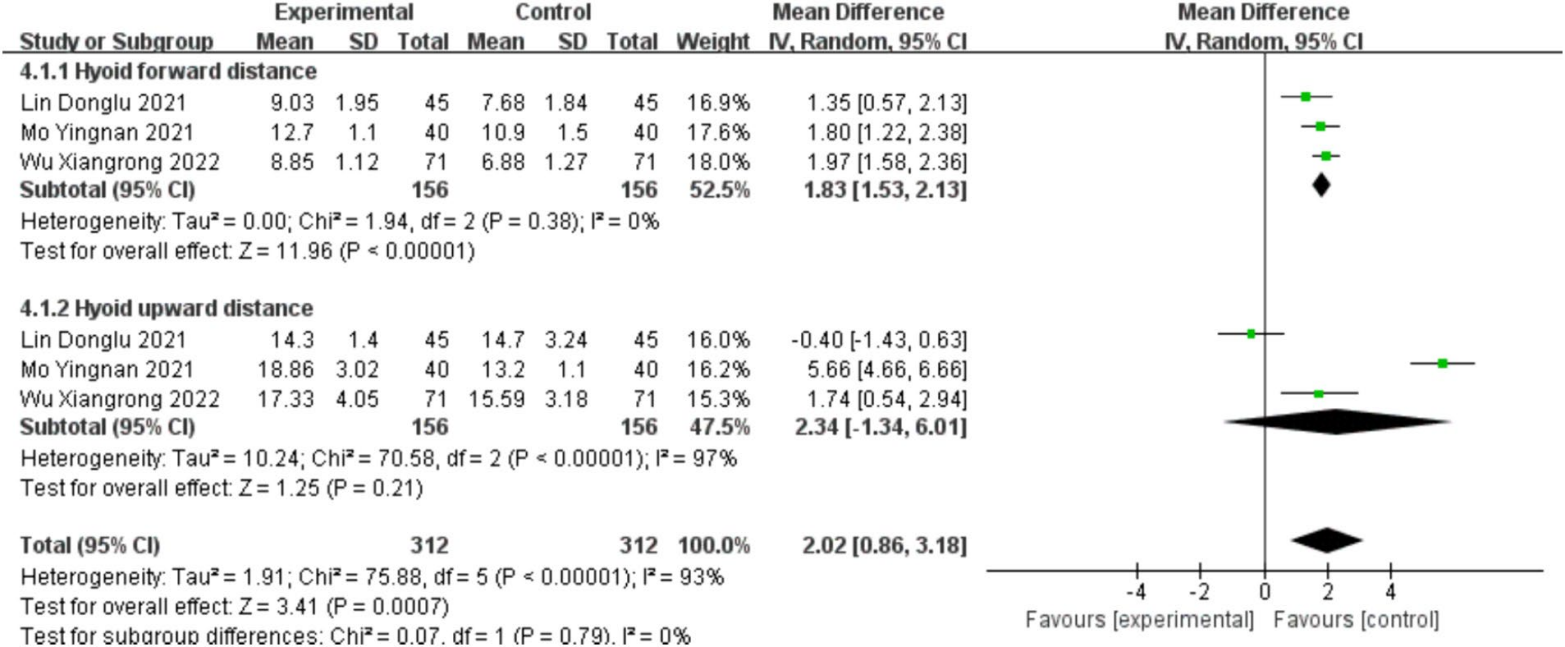
Forest plot of hyoid bone displacement.

According to the heterogeneity analysis, there was low heterogeneity in the effect sizes after treatment in the forward displacement group (*I*^2^ = 0% < 50%, and *p* = 0.38 > 0.05), while there was high heterogeneity in the effect sizes after treatment in the upward displacement group (*I*^2^ = 97% > 50%, and *p* = 0.00 < 0.05). The overall result showed high heterogeneity (*I*^2^ = 93% > 50%, and *p* = 0.00 < 0.05). Therefore, a random-effects model was used for the meta-analysis. The combined effect size was 2.02 (0.86, 3.18), and the effect size was significant (*Z* = 3.41, *p* = 0.00 < 0.05), indicating that the hyoid bone displacement in the intervention group was significantly higher than that in the control group by 2.02 mm. The intervention had a significant effect (see Figure 11).

Sensitivity analysis using the one-by-one exclusion method was performed for the studies involving hyoid bone displacement, and one study (40) was found to have a strong impact on the results (see Supplementary Figure S5).

### 3.5 Safety analysis

Four studies (39, 40, 42, 45) reported adverse events or safety evaluations related to acupuncture. One study (42) reported one case of subcutaneous hematoma and one case of mild nasal mucosal bleeding, both of which were resolved in the short term through timely treatment. The safety evaluations in three studies (39, 40, 45) did not report any adverse events.

## 4 Discussion

### 4.1 Summary of main results

This study included a total of 16 studies. The results found that compared with swallowing training, mirror therapy, urinary catheter balloon dilation, rTMS, and NMES, acupuncture alone or in combination therapy appears to be more effective in improving aspiration caused by PSD. However, caution is warranted in interpreting the results of this study. Firstly, among the included studies, 13 studies (32–38, 41–46) were identified as high risk of bias, and 3 studies (31, 39, 40) were categorized as unknown risk of bias. Secondly, in this meta-analysis, many potential variables were not disclosed, which could have varying degrees of impact on the results. This resulted in significant heterogeneity in the study. Nevertheless, it is noteworthy that the heterogeneity analysis of the primary outcome indicators in this study showed statistically significant differences, indicating the true efficacy of acupuncture for aspiration caused by PSD.

There was considerable heterogeneity in the primary outcome indicators in the studies. Subgroup analysis was attempted based on treatment duration, but the results still exhibited high heterogeneity. Subsequently, meta-regression was conducted based on publication year, intervention measures, average disease duration, and treatment course, revealing that these factors were not the cause of the high heterogeneity. Therefore, the origin of heterogeneity may be related to the type and location of stroke, as different types and locations of strokes have varying impacts on swallowing function, directly affecting the efficacy of acupuncture treatment and indirectly influencing the results of this study. Unfortunately, despite most studies reporting the number of cases of ischemic and hemorrhagic strokes, they did not separate the data when reporting results, making it impossible to verify this hypothesis. Additionally, the characteristics of acupuncture therapy itself may also contribute to the heterogeneity. The efficacy of acupuncture is influenced by factors such as acupoint selection, needle insertion time, and manipulation techniques, which are challenging to standardize across studies. Moreover, the swallowing training used in control groups in some studies is influenced by factors such as physician skill level and patient compliance, while the instrument brand and treatment intensity used in studies involving rTMS and NMES also greatly impact the study results.

Sensitivity analysis showed that apart from one study (40) having a significant impact on the analysis of hyoid bone mobility, the rest of the studies did not affect the results, possibly due to the small number of included studies. Adverse reactions to acupuncture mainly included subcutaneous hemorrhage or hematoma, without serious side effects. However, only 4 studies (39, 40, 42, 45) involved safety analysis of acupuncture treatment for post-stroke aspiration, indicating insufficient evidence regarding the safety of acupuncture treatment for aspiration caused by PSD.

In conclusion, acupuncture can be considered as a treatment method for aspiration caused by PSD. However, due to the high risk of bias and heterogeneity in the included studies, the efficacy of acupuncture treatment for aspiration caused by PSD still requires further validation.

### 4.2 Comparison with other systematic reviews

Different conclusions were found in other similar systematic reviews. A Cochrane systematic review published in 2008 (47) investigated the efficacy of acupuncture in treating acute post-stroke dysphagia and found insufficient evidence to draw any conclusions regarding the efficacy of acupuncture. However, this review only included one study with a total of 66 participants, which had a small sample size and outdated research results. More updated studies are needed to provide valuable evidence in evidence-based medicine. Another Cochrane systematic review published in 2018 (48) evaluated the efficacy of eight different treatment methods, including acupuncture. The results showed that swallowing treatment did not reduce PAS scores. However, this review did not have subgroup effects, and therefore, the results represented a general analysis of the overall efficacy of multiple treatment methods and cannot be used as independent evidence for the efficacy of acupuncture. A systematic review published in China in 2020 (49) demonstrated significant efficacy of acupuncture single therapy and acupuncture combined therapy, similar to the results of this study. However, the chosen outcome measures in that study did not include quantitative evaluation indicators for aspiration, and therefore, the research results cannot serve as evidence for the efficacy of acupuncture in treating aspiration caused by post-stroke dysphagia. Considering the results of previous systematic reviews, the efficacy of acupuncture in treating post-stroke aspiration remains controversial. The Chinese systematic review aligns with the results of this study. This may be related to the study period or the ease of including clinical randomized controlled trials conducted in China in the Chinese systematic review, as acupuncture is more widely used in China, and clinicians may have more experience and patients may have higher compliance, among other factors.

### 4.3 Mechanism studies

Normal swallowing activity consists of the oral preparatory phase, oral phase, pharyngeal phase, and esophageal phase, with the pharyngeal phase being the most critical and the stage where aspiration is most likely to occur. The key determinant of the pharyngeal phase of swallowing is the movement of the hyolaryngeal complex, which plays a crucial role in preventing food bolus entry into the airway and facilitating its entry into the esophagus (50). During the swallowing process in the pharyngeal phase, the hyoid bone and larynx undergo elevation and anterior movement. The anterior movement opens the upper esophageal sphincter, while the elevation causes epiglottic retroversion and closure of the larynx, allowing the food bolus to pass through (51). Therefore, analyzing hyoid bone displacement can provide insights into the completion of swallowing actions and the likelihood of aspiration.

Swallowing disorders result from damage to the swallowing motor area and its connection with the brainstem, leading to difficulties or inability to swallow (52–54). Although the swallowing reflex relies on the brainstem swallowing center, the initiation of swallowing is an autonomous activity (55). The swallowing neuronal network in the brainstem receives signals from the medullary swallowing center, and various cortical regions, including the insula, primary motor cortex, and somatosensory cortex, play important regulatory roles in swallowing (53). PSD is mainly caused by lesions in the swallowing cortical center, subcortical fibers, medullary swallowing center, and extrapyramidal system (2).

Acupuncture can enhance the excitability of the central nervous system, coordinate fine movements of the tongue and pharynx, and relieve pharyngeal muscle paralysis, thereby improving PSD (56). Research by Fangfang Fang and colleagues has shown that acupuncture may regulate the cortical swallowing area to the periaqueductal gray matter pathway by increasing the expression of c-Fos + neurons in the intact-side cortical swallowing area, calcium activity of neurons in the intact-side cortical swallowing area, and activity of neurons in the nucleus ambiguus, thereby achieving the therapeutic effect on PSD (57). Research by Junheng Shi has shown that in rats receiving acupuncture, a higher expression of c-Fos protein was observed in the motor neurons of the nucleus ambiguus, indicating that acupuncture can transmit impulses to the swallowing motor neurons of the nucleus ambiguus, activating the neurons and expressing immediate-early response proteins such as c-Fos. This may also be one of the central nervous system mechanisms of acupuncture in treating PSD (58).

### 4.4 Advantages and limitations

Advantages of this study: The research process strictly followed the requirements for systematic reviews and meta-analysis outlined in the Cochrane Handbook for Systematic Reviews of Interventions 6.4 (59). The study included the latest clinical randomized controlled trials, and the outcome measures relied on direct judgments from imaging examinations, avoiding subjective evaluation errors from bedside scales.

Limitations of this study: In the search strategy, to ensure comprehensive retrieval of studies, the search strategy did not include “aspiration” as a subject heading or free term. This was because it was found during the search that most studies related to acupuncture treatment for post-stroke dysphagia did not mention terms related to “aspiration” in the title or abstract. Therefore, to minimize the risk of missing studies, some sacrifices were made in the accuracy of the search strategy in this study. The high heterogeneity among the included studies made it difficult to identify the source through subgroup analysis and meta-regression. The heterogeneity in this study arose during the establishment of inclusion and exclusion criteria, which is related to the characteristics of acupuncture therapy itself. Most of the included studies lacked safety assessment and recording of adverse events, and did not provide explanations for sample size calculation methods and processes. Additionally, all the literature included in this study was published in Chinese, so the conclusions drawn need to be interpreted cautiously, as they may only indicate the efficacy of acupuncture combined therapy and acupuncture single therapy for treating aspiration caused by post-stroke dysphagia in China.

### 4.5 Implications for future research

Future research should provide more detailed reporting of experimental design, safety evaluations, sample size calculations, and other aspects to improve research quality. Case selection can be further refined, such as including only patients with a specific type of stroke to enhance the study’s specificity. Higher-quality efficacy evaluation indicators with stronger evidence should be chosen to minimize errors resulting from indirect and subjective evaluations. More clinical randomized controlled trials on post-stroke aspiration, especially for silent aspiration, should be conducted to provide a foundation for higher-quality evidence in evidence-based medicine.

## 5 Conclusion

Both acupuncture combined therapy and acupuncture single therapy can effectively improve aspiration caused by post-stroke dysphagia, with a low incidence of adverse events. However, due to the low quality of the included literature, more high-quality randomized controlled trials are still needed to confirm the effectiveness and safety of acupuncture in the treatment of aspiration caused by post-stroke dysphagia.

## Data availability statement

The original contributions presented in the study are included in the article/Supplementary material, further inquiries can be directed to the corresponding author.

## Author contributions

HL: Data curation, Formal analysis, Investigation, Software, Visualization, Writing – original draft, Writing – review & editing. JL: Conceptualization, Data curation, Resources, Supervision, Writing – review & editing. XW: Conceptualization, Methodology, Supervision, Writing – review & editing. ZZ: Conceptualization, Formal analysis, Funding acquisition, Investigation, Supervision, Writing – review & editing.
