# Supplementary material for: A systematic review and meta-analysis of acupuncture in aspiration caused by post-stroke dysphagia
Source: Front Neurol. 2024 Jun 10;15:1305056. doi: 10.3389/fneur.2024.1305056 (PMC11194430; doi:10.3389/fneur.2024.1305056)
Supplement: Supplementary file 1 [file Data_Sheet_1.docx]

**Appendix 2: Search strategy for each database**

**CBM**

(("随机对照实验"[常用字段:智能] OR "随机对照研究"[常用字段:智能] OR "RCT"[常用字段:智能] OR "随机"[常用字段:智能] OR "随机对照"[常用字段:智能]) OR (随机对照试验)) AND (("针灸"[常用字段:智能] OR "电针"[常用字段:智能] OR "耳针"[常用字段:智能] OR "体针"[常用字段:智能] OR "头针"[常用字段:智能] OR "头皮针"[常用字段:智能]) OR (针刺)) AND ((("吞咽功能障碍"[常用字段:智能] OR "咽下障碍"[常用字段:智能] OR "吞咽困难"[常用字段:智能] OR "食管咽下困难"[常用字段:智能]) OR (吞咽障碍)) AND (("CVA"[常用字段:智能] OR "CVAs"[常用字段:智能] OR "脑血管中风"[常用字段:智能] OR "中风，急性"[常用字段:智能] OR "急性脑血管意外"[常用字段:智能] OR "急性脑卒中"[常用字段:智能]) OR ("中风"[常用字段:智能] OR "脑卒中"[常用字段:智能] OR "缺血性脑卒中"[常用字段:智能] OR "脑梗死"[常用字段:智能] OR "出血性脑卒中"[常用字段:智能] OR "脑出血"[常用字段:智能] OR "急性卒中"[常用字段:智能] OR "脑中风"[常用字段:智能] OR "脑血管意外"[常用字段:智能]) OR ("卒中"[常用字段:智能])))

CNKI

（主题：卒中 + 脑卒中 + 出血性卒中 + 脑梗死(精确)）OR（主题：中风 + 出血性中风 + 缺血性脑卒中 + 出血性脑卒中 + 脑出血 + 急性卒中 + 脑中风 + 脑血管意外 + CVA + CVAs + 脑血管中风 + 中风，急性 + 急性脑血管意外 + 急性脑卒中(精确)） AND （（主题：吞咽障碍 + 吞咽功能障碍 + 咽下障碍 + 吞咽困难 + 食管咽下障碍(精确)）） AND （（主题：针刺 + 针灸 + 电针 + 耳针 + 体针 + 头针 + 头皮针(精确)）） AND （（摘要：随机对照试验）OR（摘要：随机对照实验）OR（摘要：随机对照研究）OR（摘要：RCT）OR（摘要：随机对照）OR（摘要：随机））

Wanfang

主题:(卒中 or 中风 or 脑卒中 or 缺血性脑卒中 or 脑梗死 or 出血性脑卒中 or 脑出血 or 急性卒中 or 脑中风 or 脑血管意外 or CVA or CVAs or 脑血管中风 or 中风，急性 or 急性脑血管意外 or 急性脑卒中) and 主题:(吞咽障碍 or 吞咽功能障碍 or 咽下障碍 or 吞咽困难 or 食管咽下困难) and 主题:(针刺 or 针灸 or 电针 or 耳针 or 体针 or 头针 or 头皮针) and 主题:(随机对照试验 or 随机对照实验 or 随机对照研究 or RCT or 随机对照 or 随机)

VIP

((((((((((((((((((题名或关键词=中风 OR 题名或关键词=脑卒中) OR 题名或关键词=缺血性脑卒中) OR 题名或关键词=脑梗死) OR 题名或关键词=出血性脑卒中) OR 题名或关键词=脑出血) OR 题名或关键词=急性卒中) OR 题名或关键词=脑中风) OR 题名或关键词=脑血管意外) OR 题名或关键词=CVA) OR 题名或关键词=CVAs) OR 题名或关键词=脑血管中风) OR 题名或关键词=中风，急性) OR 题名或关键词=急性脑血管意外) OR 题名或关键词=急性脑卒中) OR 题名或关键词=卒中) AND ((((题名或关键词=吞咽功能障碍 OR 题名或关键词=咽下障碍) OR 题名或关键词=吞咽困难) OR 题名或关键词=食管咽下困难) OR 题名或关键词=吞咽障碍)) AND ((((((题名或关键词=针灸 OR 题名或关键词=电针) OR 题名或关键词=耳针) OR 题名或关键词=体针) OR 题名或关键词=头针) OR 题名或关键词=头皮针) OR 题名或关键词=针刺)) AND (((((摘要=随机对照实验 OR 摘要=随机对照研究) OR 摘要=RCT) OR 摘要=随机对照) OR 摘要=随机) OR 摘要=随机对照试验))

PUBMED

(((randomized controlled trial[Publication Type] OR randomized[Title/Abstract] OR placebo[Title/Abstract]) AND ((stroke) OR ((((((((((((Strokes[Title/Abstract]) OR (Cerebrovascular Accident[Title/Abstract])) OR (Cerebrovascular Accidents[Title/Abstract])) OR (CVA (Cerebrovascular Accident[Title/Abstract]))) OR (CVAs (Cerebrovascular Accident[Title/Abstract]))) OR (Cerebrovascular Apoplexy[Title/Abstract])) OR (Apoplexy, Cerebrovascular[Title/Abstract])) OR (Vascular Accident, Brain[Title/Abstract])) OR (Brain Vascular Accident[Title/Abstract])) OR (Brain Vascular Accidents[Title/Abstract])) OR (Vascular Accidents, Brain[Title/Abstract])) OR (Cerebrovascular Stroke[Title/Abstract])))) AND (("Deglutition Disorders"[Mesh]) OR (((((((((Deglutition Disorder[Title/Abstract]) OR (Disorders, Deglutition[Title/Abstract])) OR (Swallowing Disorders[Title/Abstract])) OR (Swallowing Disorder[Title/Abstract])) OR (Dysphagia[Title/Abstract])) OR (Oropharyngeal Dysphagia[Title/Abstract])) OR (Dysphagia, Oropharyngeal[Title/Abstract])) OR (Esophageal Dysphagia[Title/Abstract])) OR (Dysphagia, Esophageal[Title/Abstract])))) AND (("Acupuncture"[Mesh]) OR ((((((((((((Pharmacopuncture[Title/Abstract]) OR (electroacupuncture[Title/Abstract])) OR (Acupuncture, Ear[Title/Abstract])) OR (Acupunctures, Ear[Title/Abstract])) OR (Ear Acupunctures[Title/Abstract])) OR (Auricular Acupuncture[Title/Abstract])) OR (Ear Acupuncture[Title/Abstract])) OR (Acupuncture, Auricular[Title/Abstract])) OR (Acupunctures, Auricular[Title/Abstract])) OR (Auricular Acupunctures[Title/Abstract])) OR (scalp acupuncture[Title/Abstract])) OR (body acupuncture[Title/Abstract])))

EMBASE

#1

'stroke'/exp OR stroke

#2

'strokes':ab,ti OR 'cerebrovascular accident':ab,ti OR 'cerebrovascular accidents':ab,ti OR 'cva (cerebrovascular accident)':ab,ti OR 'cvas (cerebrovascular accident)':ab,ti OR 'cerebrovascular apoplexy':ab,ti OR 'apoplexy, cerebrovascular':ab,ti OR 'vascular accident, brain':ab,ti OR 'brain vascular accident':ab,ti OR 'brain vascular accidents':ab,ti OR 'vascular accidents, brain':ab,ti OR 'cerebrovascular stroke':ab,ti OR 'cerebrovascular strokes':ab,ti OR 'stroke, cerebrovascular':ab,ti OR 'strokes, cerebrovascular':ab,ti OR 'apoplexy':ab,ti OR 'cerebral stroke':ab,ti OR 'cerebral strokes':ab,ti OR 'stroke, cerebral':ab,ti OR 'strokes, cerebral':ab,ti OR 'stroke, acute':ab,ti OR 'acute stroke':ab,ti OR 'acute strokes':ab,ti OR 'strokes, acute':ab,ti OR 'cerebrovascular accident, acute':ab,ti OR 'acute cerebrovascular accident':ab,ti OR 'acute cerebrovascular accidents':ab,ti OR 'cerebrovascular accidents, acute':ab,ti

#3

#1 OR #2

#4

deglutition AND disorders

#5

'deglutition disorder':ab,ti OR 'disorders, deglutition':ab,ti OR 'swallowing disorders':ab,ti OR 'swallowing disorder':ab,ti OR 'dysphagia':ab,ti OR 'oropharyngeal dysphagia':ab,ti OR 'dysphagia, oropharyngeal':ab,ti OR 'esophageal dysphagia':ab,ti OR 'dysphagia, esophageal':ab,ti

#6

#4 OR #5

#7

Acupuncture

#8

'pharmacopuncture':ab,ti OR 'electroacupuncture':ab,ti OR 'acupuncture, ear':ab,ti OR 'acupunctures, ear':ab,ti OR 'ear acupunctures':ab,ti OR 'auricular acupuncture':ab,ti OR 'ear acupuncture':ab,ti OR 'acupuncture, auricular':ab,ti OR 'acupunctures, auricular':ab,ti OR 'auricular acupunctures':ab,ti OR 'scalp acupuncture':ab,ti OR 'body acupuncture':ab,ti

#9

#7 OR #8

#10

'randomized controlled trial':ab,ti OR 'randomized':ab,ti OR 'placebo':ab,ti OR 'rct':ab,ti

#11

#3 AND #6 AND #9 AND #10

WOS

#1

(((((((((((((((((((((((((((TS=(stroke)) OR TS=(Strokes)) OR TS=(Cerebrovascular Accident)) OR TS=(Cerebrovascular Accidents)) OR TS=(CVA (Cerebrovascular Accident))) OR TS=(CVAs (Cerebrovascular Accident))) OR TS=(Cerebrovascular Apoplexy)) OR TS=(Apoplexy, Cerebrovascular)) OR TS=(Vascular Accident, Brain)) OR TS=(Brain Vascular Accident)) OR TS=(Brain Vascular Accidents)) OR TS=(Vascular Accidents, Brain)) OR TS=(Cerebrovascular Stroke)) OR TS=(Cerebrovascular Strokes)) OR TS=(Stroke, Cerebrovascular)) OR TS=(Strokes, Cerebrovascular)) OR TS=(Apoplexy)) OR TS=(Cerebral Stroke)) OR TS=(Cerebral Strokes)) OR TS=(Stroke, Cerebral)) OR TS=(Strokes, Cerebral)) OR TS=(Stroke, Acute)) OR TS=(Acute Stroke)) OR TS=(Strokes, Acute)) OR TS=(Cerebrovascular Accident, Acute)) OR TS=(Acute Cerebrovascular Accident)) OR TS=(Acute Cerebrovascular Accidents)) OR TS=(Cerebrovascular Accidents, Acute)

#2

(((((((((TS=(Deglutition Disorders)) OR TS=(Deglutition Disorder)) OR TS=(Disorders, Deglutition)) OR TS=(Swallowing Disorders)) OR TS=(Swallowing Disorder)) OR TS=(Dysphagia)) OR TS=(Oropharyngeal Dysphagia)) OR TS=(Dysphagia, Oropharyngeal)) OR TS=(Esophageal Dysphagia)) OR TS=(Dysphagia, Esophageal)

#3

((((((((((((TS=(acupuncture)) OR TS=(Pharmacopuncture)) OR TS=(electroacupuncture)) OR TS=(Acupuncture, Ear)) OR TS=(Acupunctures, Ear)) OR TS=(Ear Acupunctures)) OR TS=(Auricular Acupuncture)) OR TS=(Ear Acupuncture)) OR TS=(Acupuncture, Auricular)) OR TS=(Acupunctures, Auricular)) OR TS=(Auricular Acupunctures)) OR TS=(scalp acupuncture)) OR TS=(body acupuncture)

#4

(((TS=(randomized controlled trial)) OR TS=(randomized)) OR TS=(placebo)) OR TS=(RCT)

#5

#1 AND #2 AND #3 AND #4

Cochrane Library

#1

stroke

#2

(Strokes):ab,ti,kw OR (Cerebrovascular Accident):ab,ti,kw OR (Cerebrovascular Accidents):ab,ti,kw OR (CVA (Cerebrovascular Accident)):ab,ti,kw OR (CVAs (Cerebrovascular Accident)):ab,ti,kw OR (Cerebrovascular Apoplexy):ab,ti,kw OR (Apoplexy, Cerebrovascular):ab,ti,kw OR (Vascular Accident, Brain):ab,ti,kw OR (Brain Vascular Accident):ab,ti,kw OR (Brain Vascular Accidents):ab,ti,kw OR (Vascular Accidents, Brain):ab,ti,kw OR (Cerebrovascular Stroke):ab,ti,kw OR (Cerebrovascular Strokes):ab,ti,kw OR (Stroke, Cerebrovascular):ab,ti,kw OR (Strokes, Cerebrovascular):ab,ti,kw OR (Apoplexy):ab,ti,kw OR (Cerebral Stroke):ab,ti,kw OR (Cerebral Strokes):ab,ti,kw OR (Stroke, Cerebral):ab,ti,kw OR (Strokes, Cerebral):ab,ti,kw OR (Stroke, Acute):ab,ti,kw OR (Acute Stroke):ab,ti,kw OR (Acute Strokes):ab,ti,kw OR (Strokes, Acute):ab,ti,kw OR (Cerebrovascular Accident, Acute):ab,ti,kw OR (Acute Cerebrovascular Accident):ab,ti,kw OR (Acute Cerebrovascular Accidents):ab,ti,kw OR (Cerebrovascular Accidents, Acute):ab,ti,kw

#3

#1 OR #2

#4

Deglutition Disorders

#5

(Deglutition Disorder):ab,ti,kw OR (Disorders, Deglutition):ab,ti,kw OR (Swallowing Disorders):ab,ti,kw OR (Swallowing Disorder):ab,ti,kw OR (Dysphagia):ab,ti,kw OR (Oropharyngeal Dysphagia):ab,ti,kw OR (Dysphagia, Oropharyngeal):ab,ti,kw OR (Esophageal Dysphagia):ab,ti,kw OR (Dysphagia, Esophageal):ab,ti,kw

#6

#4 OR #5

#7

acupuncture

#8

(Pharmacopuncture):ab,ti,kw OR (electroacupuncture):ab,ti,kw OR (Acupuncture, Ear):ab,ti,kw OR (Acupunctures, Ear):ab,ti,kw OR (Ear Acupunctures):ab,ti,kw OR (Auricular Acupuncture):ab,ti,kw OR (Ear Acupuncture):ab,ti,kw OR (Acupuncture, Auricular):ab,ti,kw OR (Acupunctures, Auricular):ab,ti,kw OR (Auricular Acupunctures):ab,ti,kw OR (scalp acupuncture):ab,ti,kw OR (body acupuncture):ab,ti,kw

#9

#7 OR #8

#10

(randomized controlled trial):ab,ti,kw OR (randomized):ab,ti,kw OR (placebo):ab,ti,kw OR (rct):ab,ti,kw

#11

#3 AND #6 AND #9 AND #10
