# Supplementary figures and images for: A systematic review and meta-analysis of acupuncture in aspiration caused by post-stroke dysphagia
Source: Front Neurol. 2024 Jun 10;15:1305056. doi: 10.3389/fneur.2024.1305056 (PMC11194430; doi:10.3389/fneur.2024.1305056)

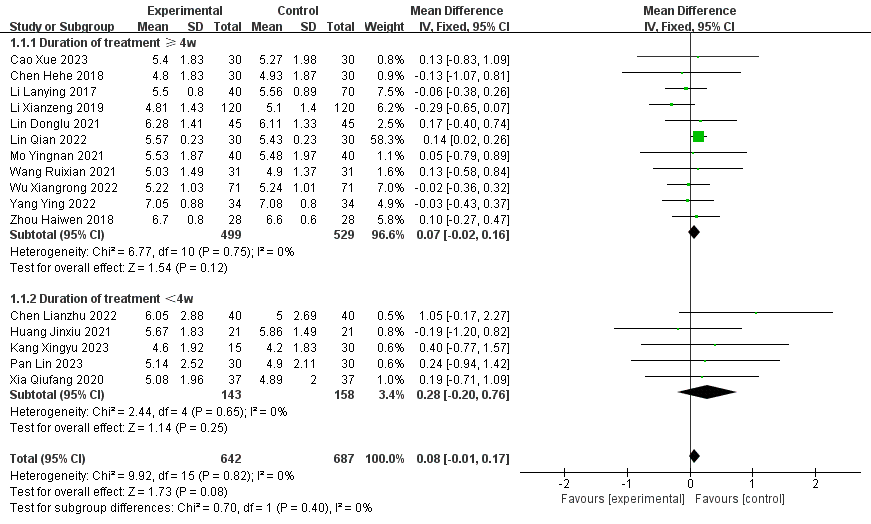

Supplement: Supplementary file 2 [file Image_1.png]

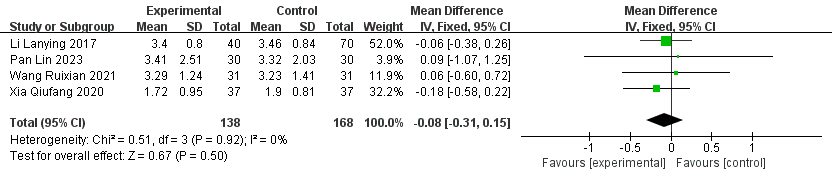

Supplement: Supplementary file 3 [file Image_2.png]

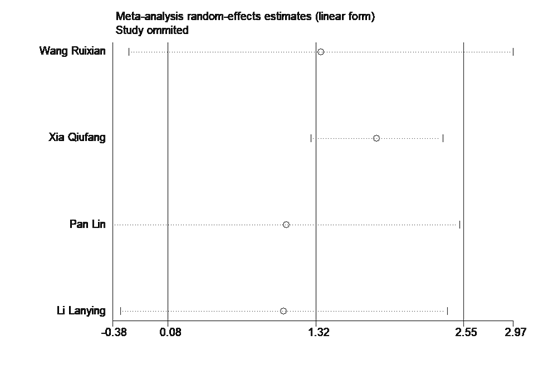

Supplement: Supplementary file 4 [file Image_3.png]

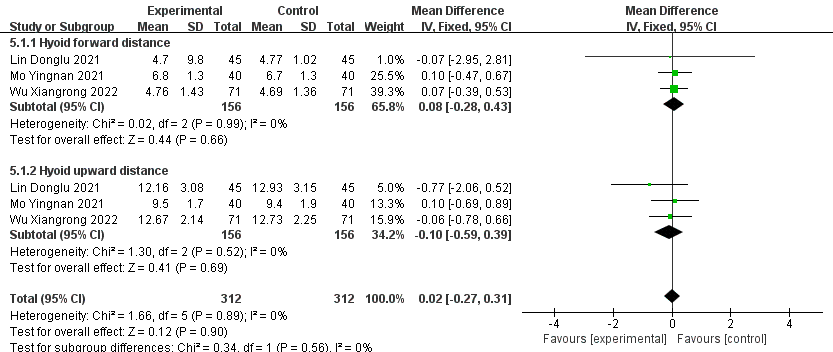

Supplement: Supplementary file 5 [file Image_4.png]

Meta-analysis fixed-effects estimates (linear form)

Study omitted

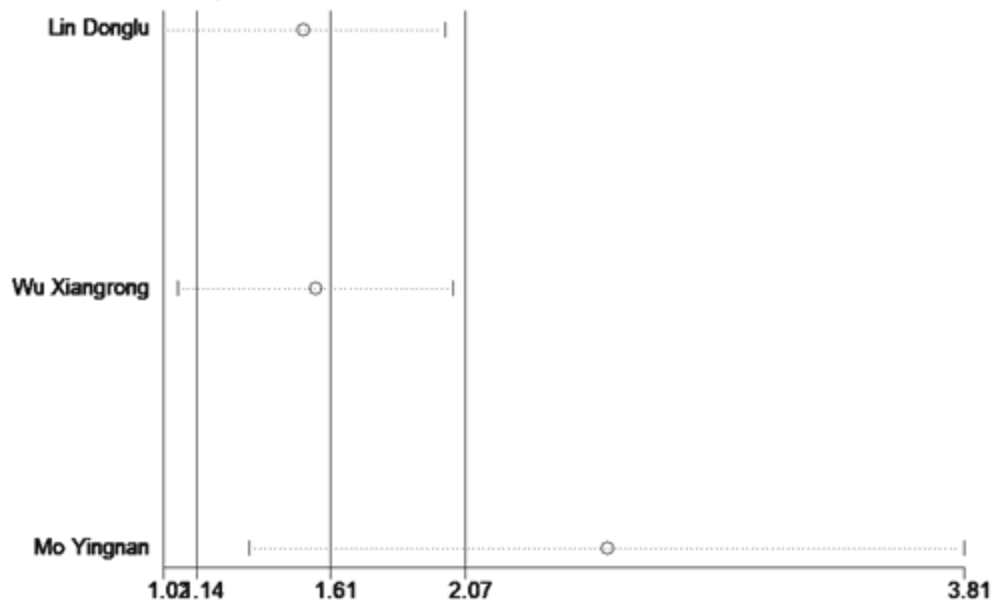

Supplement: Supplementary file 6 [file Image_5.pdf]
